# A Colorimetric Strategy for Arsenic(V) Determination in Rice via Boosting the Peroxidase-like Activity of NCuzyme

**DOI:** 10.3390/molecules31193494

**Published:** 2026-10-01

**Authors:** Lei Xu, Mao-Ling Luo, Shan Qiu, Jing-Jing Dai, Huan Zhu, Feng-Qing Yang

**Affiliations:** School of Chemistry and Chemical Engineering, Chongqing University, Chongqing 401331, China; 20195277@cqu.edu.cn (L.X.); 20185486@cqu.edu.cn (M.-L.L.); 202418021112t@stu.cqu.edu.cn (S.Q.); 202318021129t@stu.cqu.edu.cn (J.-J.D.); 202318021131t@stu.cqu.edu.cn (H.Z.)

**Keywords:** NCuzyme, peroxidase-like activity, colorimetric detection, As(V), rice sample

## Abstract

Arsenic (As) is extremely poisonous, and prolonged exposure greatly raises the likelihood of developing cancer. It is crucial to develop selective and sensitive methods for detecting As. In this study, using melamine and Cu(II), a Cu-based complex (NCuzyme) with peroxidase (POD)-like activity was prepared using a green and simple approach for the first time. NCuzyme can catalyze the oxidation of 3,3′,5,5′-tetramethylbenzidine (TMB) with H_2_O_2_ to form a blue product (oxTMB). The addition of As(V) can boost the POD-like activity of NCuzyme, deepening the blue color and increasing the absorbance signal of the reaction system. Therefore, a colorimetric method has been developed for the detection of As(V). This method exhibits excellent selectivity for As(V), with a linear range of 4–150 μg/L and a limit of detection of 3.35 μg/L, which is below the limit for arsenic in drinking water (10 μg/L) set by the World Health Organization. Finally, the developed colorimetric sensing method has been successfully applied to detect As(V) in real sample (rice), with good recovery rates (93.3–102.9%) and an RSD of less than 2.3%. In summary, the colorimetric method developed based on enhancing POD-like activity of NCuzyme provides a rapid and simple method for the As(V) detection.

## 1. Introduction

Arsenic (As) is one of the most toxic metalloid elements that can enter the human body primarily through contaminated groundwater and food [1,2]. The toxicity of As is closely related to its chemical form. Inorganic arsenic is highly toxic compared to organic arsenic, although As(III) is more toxic than As(V), the As(V) form is more stable than As(III) [3,4]. As exhibits various toxic effects, including cardiovascular, neurotoxic, and nephrotoxic effects [5,6]. Long-term excessive exposure to As can lead to damage to multiple organs and systems, and significantly increases the risk of malignant tumors, such as lung cancer, skin cancer, and bladder cancer [7]. The limit for arsenic in drinking water has been set at 10 μg/L by the World Health Organization (WHO). Therefore, it is of great significance to develop methods for the detection of As(V) with high sensitivity and selectivity.

Currently, the main methods for detecting As include instrumental analytical techniques, including atomic absorption spectrometry [8], inductively coupled plasma mass spectrometry [9,10,11], and atomic fluorescence spectrometry [12,13]. Although these methods offer high sensitivity and good accuracy, they typically require expensive equipment, complex sample preparation, and specialized operators, making it difficult to meet the demands of rapid on-site detection [14,15,16]. Alternatively, colorimetric methods have attracted widespread attentions in the field of rapid detection due to their low cost, simplicity, and visual nature [17]. For instance, Phosri et al. [18] developed Fe(III)-assisted tartrate-stabilized AuNPs (Tar-AuNPs-Fe(III)) for As(III) detection, achieving a LOD of 21 μg/L with a visible red-to-blue color change. Additionally, colorimetric sensing strategies based on nanozymes offer a new approach to the on-site detection of heavy metal ions, owing to their adjustable catalytic activity, high stability, and easily modifiable nature [19,20,21]. Wang et al. [22] developed a Mn-urea material with As(V)-enhanced oxidase (OXD)-like activity, which was successfully employed for the colorimetric detection of inorganic arsenic selectively. Furthermore, the hydrolysis of ascorbic acid 2-phosphate (AAP) can be catalyzed by acid phosphatase (ACP) to produce ascorbic acid (AA), while the catalytic activity of ACP can be inhibited by As(V) [23]. Building on this, previous studies have utilized mimetic enzymes and ACP to construct enzyme cascade reaction systems for the detection of As(V). For instance, Xu et al. [24] prepared a multifunctional Zn-based MOF material loaded with ACP and hemin (ACP/hemin@Zn-MOF), enabling ratio fluorescence detection of As(V). In addition, a colorimetric method has been developed by Wang et al. [25] to detect As(V) by utilizing the cerium (Ce) coordination polymer nanoparticles with OXD-like activity and introduction of ACP and AAP. However, such methods typically involve the use of natural enzymes and are subject to limitations like poor stability, high cost, and stringent storage conditions.

As a nitrogen-rich organic ligand, the molecule of melamine (MEL) contains three amino groups and a triazine ring, providing abundant metal coordination sites that facilitate the creation of stable coordination structures with metal ions. At the same time, its high nitrogen content helps to regulate the electronic property and catalytic performance of the material [26,27]. Chai et al. [28] successfully synthesized a Cu-MOF with abundant Cu–N coordination centers using MEL as a linker via a conventional hydrothermal method, exhibiting excellent laccase-like activity.

Inspired by these works, MEL was selected as the organic ligand to coordinate with Cu, aiming to construct a Cu-MEL composite with abundant Cu–N coordination sites and tunable nanozyme activity for the colorimetric detection of As. Based on this, the study synthesized a novel Cu-based nanozyme (NCuzyme) with peroxidase (POD)-like activity. Furthermore, capitalizing on the boosting effect of As(V) on the POD-like activity of NCuzyme, a colorimetric detection method for As(V) was developed and applied in the analysis of rice sample.

## 2. Results and Discussion

### 2.1. Characterization of NCuzyme

**Figure 1:**
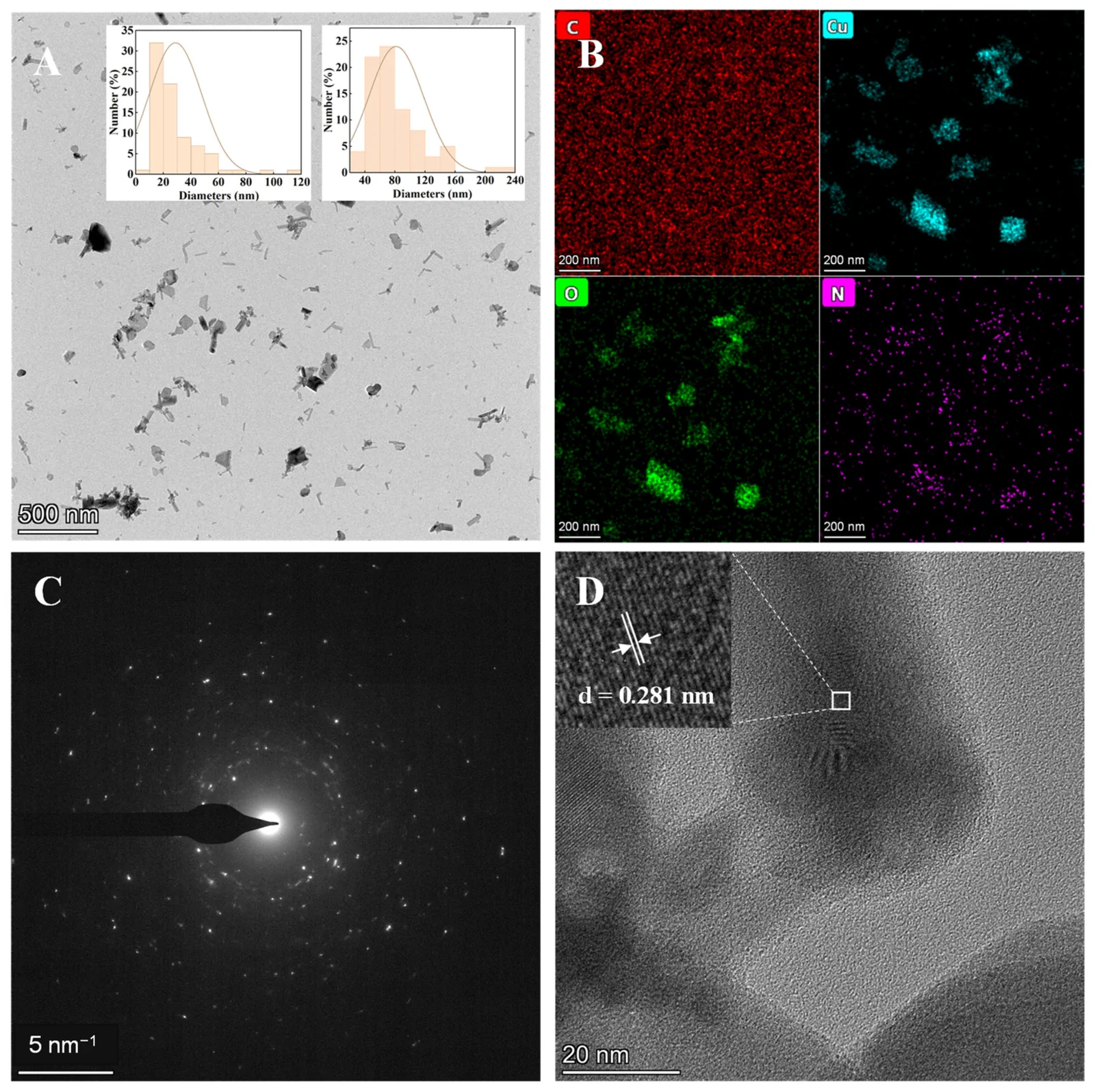
(**A**) TEM image (inset: particle size distribution), (**B**) EDX mapping images, (**C**) SAED pattern, and (**D**) HRTEM image of NCuzyme.

**Figure 2:**
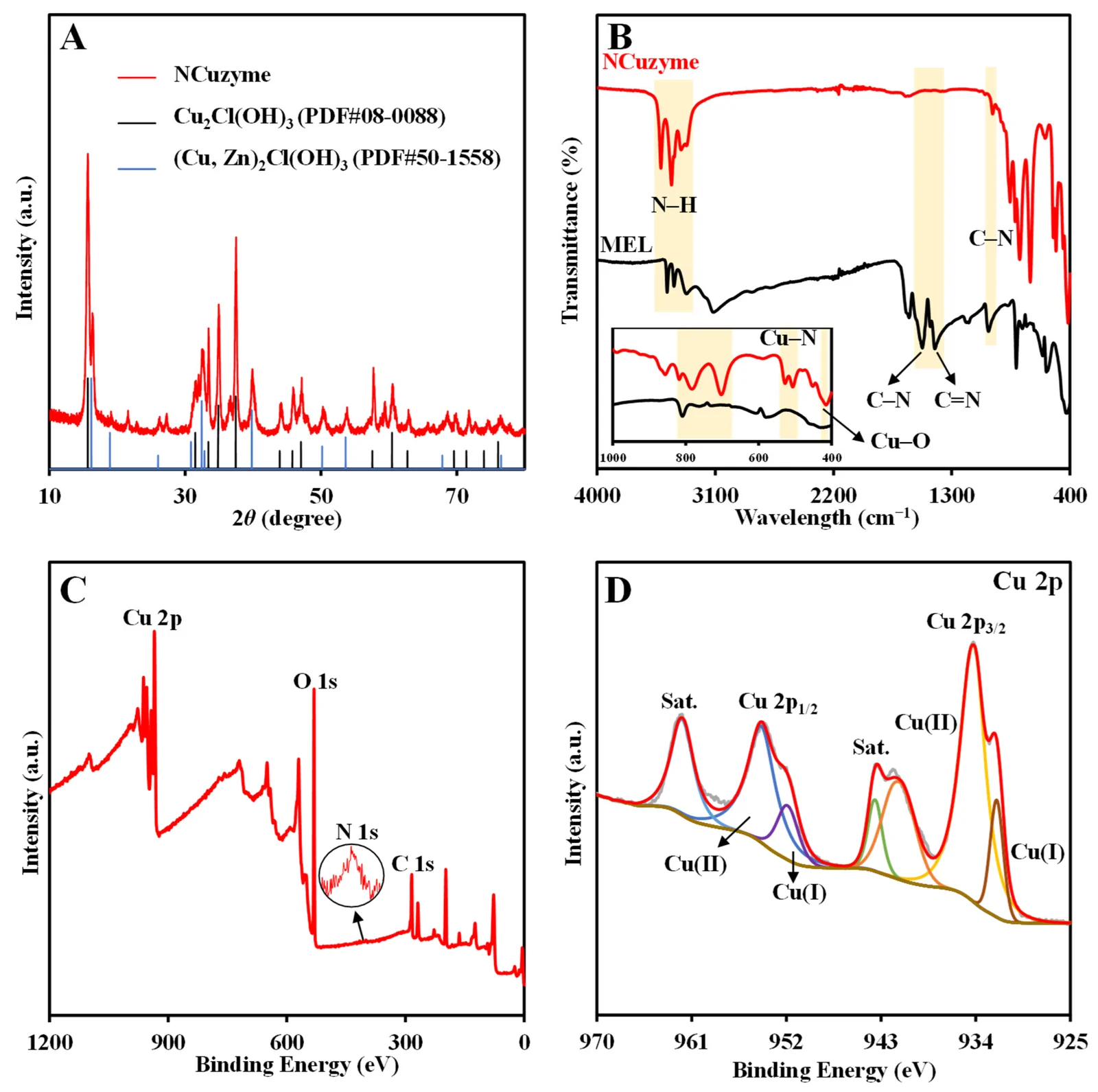
(**A**) XRD pattern of NCuzyme; (**B**) FT-IR spectra of NCuzyme and MEL (inset: enlarged image of 400–1000 cm^−1^); XPS spectra of NCuzyme: (**C**) survey spectrum, (**D**) Cu 2p.

The as-prepared NCuzyme was characterized by transmission electron microscopy (TEM) for its morphology and structure. As shown in Figure 1A, the synthesized NCuzyme exhibits irregular nanosheets with average width and length of about 29 nm and 82 nm, respectively (inset in Figure 1A). The energy dispersive X-ray spectroscopy (EDX) results (Figure 1B) confirm that four elements like carbon (C), oxygen (O), nitrogen (N), and copper (Cu) are distributed evenly on the NCuzyme’s surface. The selected-area electron diffraction (SAED) pattern (Figure 1C) reveals relatively distinct concentric diffraction rings, indicating that the NCuzyme has a polycrystalline structure. In addition, the inset graph from the high-resolution transmission electron microscopy (HRTEM) image (Figure 1D) shows clearly the lattice spacing of 0.281 nm corresponding to the (200) lattice plane of Cu_2_Cl(OH)_3_. The X-ray diffraction (XRD) results (Figure 2A) show that the NCuzyme exhibits clear diffraction peaks, demonstrating a high degree of crystallinity. The positions of the diffraction peaks are essentially consistent with those of the standard samples Cu_2_Cl(OH)_3_ (PDF#08-0088) and (Cu, Zn)_2_Cl(OH)_3_ (PDF#50-1558). Therefore, the structure of NCuzyme is like atacamite-type layered crystals.

Fourier transform infrared spectroscopy (FT-IR) spectra were analyzed to investigate the functional groups of NCuzyme during the synthesis. The FT-IR spectrum of NCuzyme (Figure 2B) exhibits significant changes compared with that of MEL. The absorption peaks attributed to N–H stretching vibrations (3318–3466 cm^−1^) shift towards higher wavenumbers, indicating an interaction between Cu and the amino group. Concurrently, the characteristic peaks attributed to the C–N and C=N stretching vibrations of the triazine ring in MEL (1524 cm^−1^ and 1429 cm^−1^) have completely disappeared, and the peak corresponding to the C–N stretching vibration between the triazine ring and the exocyclic amino group (1020 cm^−1^) has significantly weakened, indicating that the conjugated structure of the triazine ring has been severely disrupted. However, the out-of-plane bending vibration of the triazine ring remains but is shifted from 809 cm^−1^ to 783 cm^−1^. Additionally, the absorption peak at 702 cm^−1^, which may be attributed to the deformation vibration of the triazine ring skeleton, has markedly enhanced. These indicate that the ring skeleton structure of MEL has been retained after the reaction and coordinates with Cu. Furthermore, new peaks at 510 cm^−1^ (Cu–N) and 415 cm^−1^ (Cu–O) correspond to their stretching vibrations. These results further confirm the successful preparation of NCuzyme.

X-ray photoelectron spectrometry (XPS) was used to further analyze the chemical state and elemental composition of NCuzyme. The full-scan survey spectrum (Figure 2C) confirms the existence of the four elements like C, O, N, and Cu in NCuzyme. The Cu 2p spectrum (Figure 2D) displays two main peaks (Cu 2p_1/2_ and Cu 2p_3/2_) along with two satellite peaks. The peaks at 934.14 eV and 954.14 eV correspond to Cu(II), while those at 932.00 eV and 951.72 eV correspond to Cu(I), indicating the presence of multivalent Cu in NCuzyme. The C 1s spectrum (Figure S1A) was fitted with two peaks attributed to C–C (284.80 eV) and C–N (288.33 eV), respectively. The N 1s spectrum (Figure S1B) was deconvoluted into two peaks at 400.53 and 399.10 eV attributed to Cu–N and C–N, respectively. In addition, three peaks in the O 1s spectrum (Figure S1C) at 529.32 eV, 530.90 eV, and 532.21 eV correspond to Cu–O, –OH, and C–O, respectively. The results further confirm the coordination of Cu with N and O.

### 2.2. Feasibility Study and Experimental Conditions Optimization

Using 3,3′,5,5′-tetramethylbenzidine (TMB) as the substrate, the POD-like activity of NCuzyme was evaluated. Results indicate that TMB was oxidized only in the simultaneous presence of TMB, H_2_O_2_, and NCuzyme, resulting in a distinct absorption peak at 652 nm (Figure S2A). This indicates that NCuzyme possesses strong POD-like activity. Notably, As(V) can enhance the POD-like activity of NCuzyme significantly, resulting in an increase in absorbance at 652 nm. Consequently, a colorimetric method based on NCuzyme was developed for the direct As(V) detection.

Using ∆A as the evaluation criterion, a systematic investigation was conducted to optimize the key factors, such as the molar ratio of Cu(II) and MEL, and synthesis temperature and time for the synthesis of the material, the concentrations of NCuzyme and substrates, the type of buffer and pH, and reaction temperature and time of the detection conditions. Results indicate that the POD-like activity of NCuzyme gradually increased with an increased proportion of Cu(II) (Figure S2B). When the molar ratio of Cu(II) to MEL was 2:1, ∆A reached its maximum value. Therefore, 2:1 was chosen as the optimized ratio for NCuzyme synthesis. The effect of synthesis temperature is primarily related to the solubility of MEL. MEL has low solubility in cold water, and a small amount of MEL remains undissolved even at 35 °C. When the temperature was raised to 45 °C, MEL dissolved completely and ∆A increased significantly. Therefore, 45 °C was determined to be the optimized synthesis temperature (Figure S2C). Figure S2D shows that ideal detection results can be achieved with a synthesis time of 5 min, so the synthesis time was set at 5 min for the next experiments. The effect of NCuzyme concentration on the reaction system is shown in Figure S2E. A lower dilution factor of the NCuzyme suspension (i.e., a higher concentration) results in increased absorbance of the reaction system, further confirming its POD-like activity. When the dilution factor was 40-fold, ∆A reached its maximum value. Thus, the NCuzyme suspension was diluted 40-fold in subsequent experiments. Figure S2F,G show that the catalytic activity of NCuzyme increases with rising concentrations of TMB and H_2_O_2_, and plateaus at 15 mM and 10 mM, respectively. At the same time, the detection performance simultaneously reaches its optimum. The catalytic activity of the material and detection performance of the method may also be influenced by the type of buffer. As shown in Figure S2H, although the catalytic activity of NCuzyme is higher in buffers than in pure water, the ∆A value is the highest in pure water system. Consequently, pure water as the solvent for As(V) detection was selected ultimately. Furthermore, the pH of the water was adjusted using HCl and NaOH. Figure S2I shows that the catalytic activity of NCuzyme exhibits a pronounced pH dependence: the lower the pH, the higher the activity, with ∆A being greater at pH 5 or 6. It is worth noting that pure water without pH adjustment exhibits the highest ∆A. Consequently, pure water was used directly in subsequent experiments, ensuring good detection performance while simplifying the procedure. Furthermore, the optimized reaction temperature and time are 35 °C and 6 min, respectively (Figure S2J,K).

### 2.3. Colorimetric Detection of As(V)

**Figure 3:**
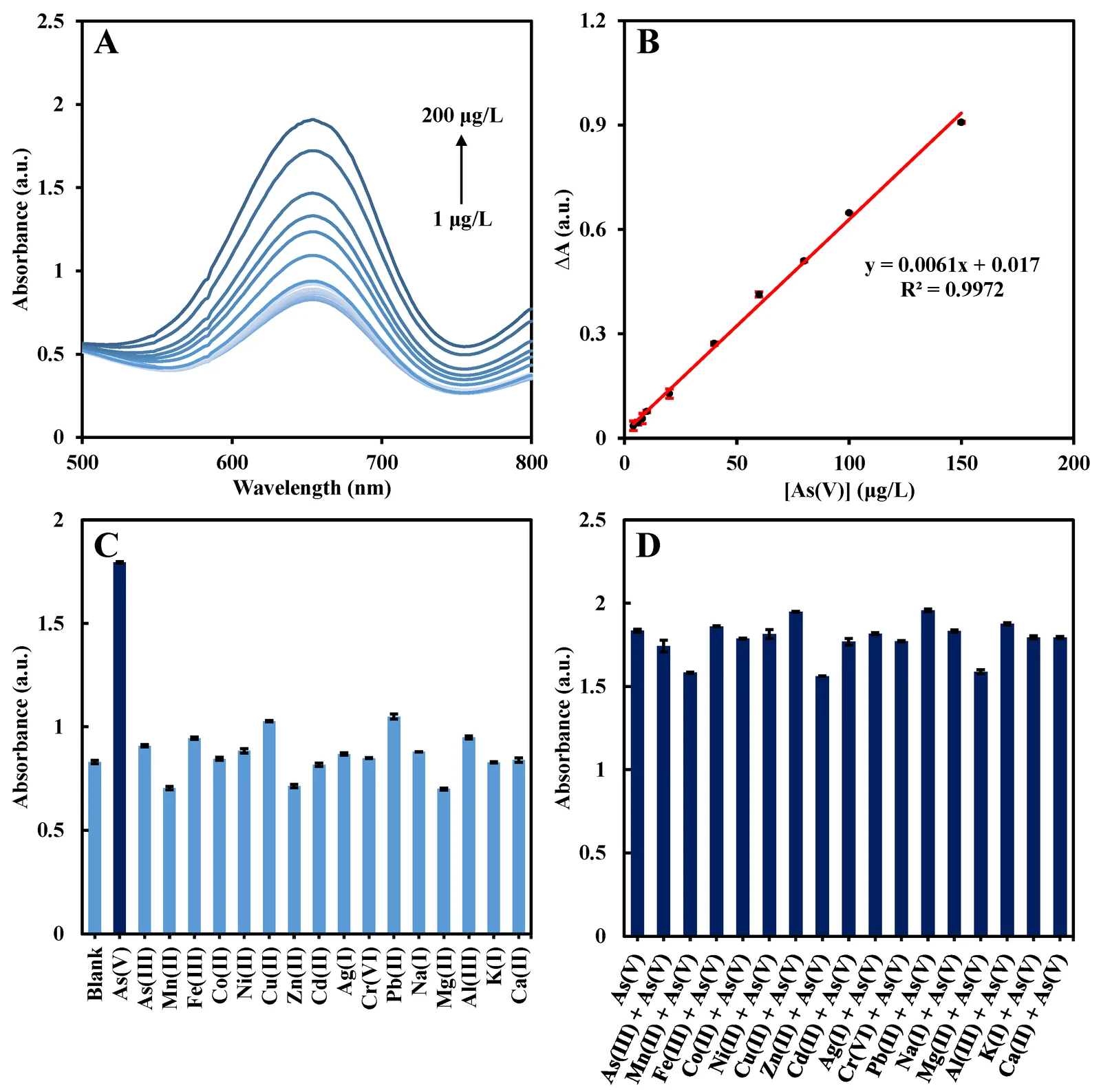
(**A**) UV-Vis absorption spectra with adding different concentrations of As(V). (**B**) Linear relationships of the As(V) concentration and absorbance. The concentrations refer to the final assay concentrations in the 1.5 mL reaction system. (**C**) Selectivity and (**D**) interference study of NCuzyme for the detection of As(V). The error bar represents three independent measurements.

The colorimetric detection of As(V) was carried out under optimized experimental conditions. As shown in Figure 3A, the absorbance at 652 nm was enhanced with the increase in As(V) concentration. Within the range of 4–150 μg/L, the absorbance and the As(V) concentration have a good linear correlation; the linear regression equation is ∆A = 0.0061 [As(V)] + 0.017 (R^2^ = 0.9972) (Figure 3B), with a limit of detection (LOD) of 3.35 μg/L. As shown in Table 1, the developed colorimetric method based on NCuzyme for As(V) detection has a low LOD, and the synthesis of the material only requires 5 min. Therefore, based on boosting the POD-like activity of NCuzyme, the effective and convenient detection of As(V) can be realized. Furthermore, to assess its suitability for practical analysis, the interference resistance and selectivity of the method were investigated. As shown in Figure 3C, most of the metal ions’ concentrations were five times that of As(V), but only As(V) can significantly enhance the detection system’s absorbance. Furthermore, the enhancement effect of As(V) was not significantly affected in the presence of other interfering ions (Figure 3D). Therefore, the colorimetric sensing method based on NCuzyme has excellent interference resistance and selectivity for As(V) detection.

### 2.4. Rice Sample Analysis

Rice is the primary dietary source of inorganic arsenic intake for humans. During their growth, rice plants readily accumulate arsenic from the soil and irrigation water, and this arsenic is difficult to remove during processing. To evaluate the feasibility of the developed colorimetric method for As(V) detection in real samples, recovery experiments were performed by spiking processed rice samples with varying concentrations of As(V). As shown in Table 2, the recovery rates for As(V) detection are in the range of 93.3–102.9%, and the relative standard deviation (RSD) is lower than 2.3%. Therefore, the developed method is suitable for As(V) detection in rice samples.

### 2.5. Study on Enhancement Mechanism

**Figure 4:**
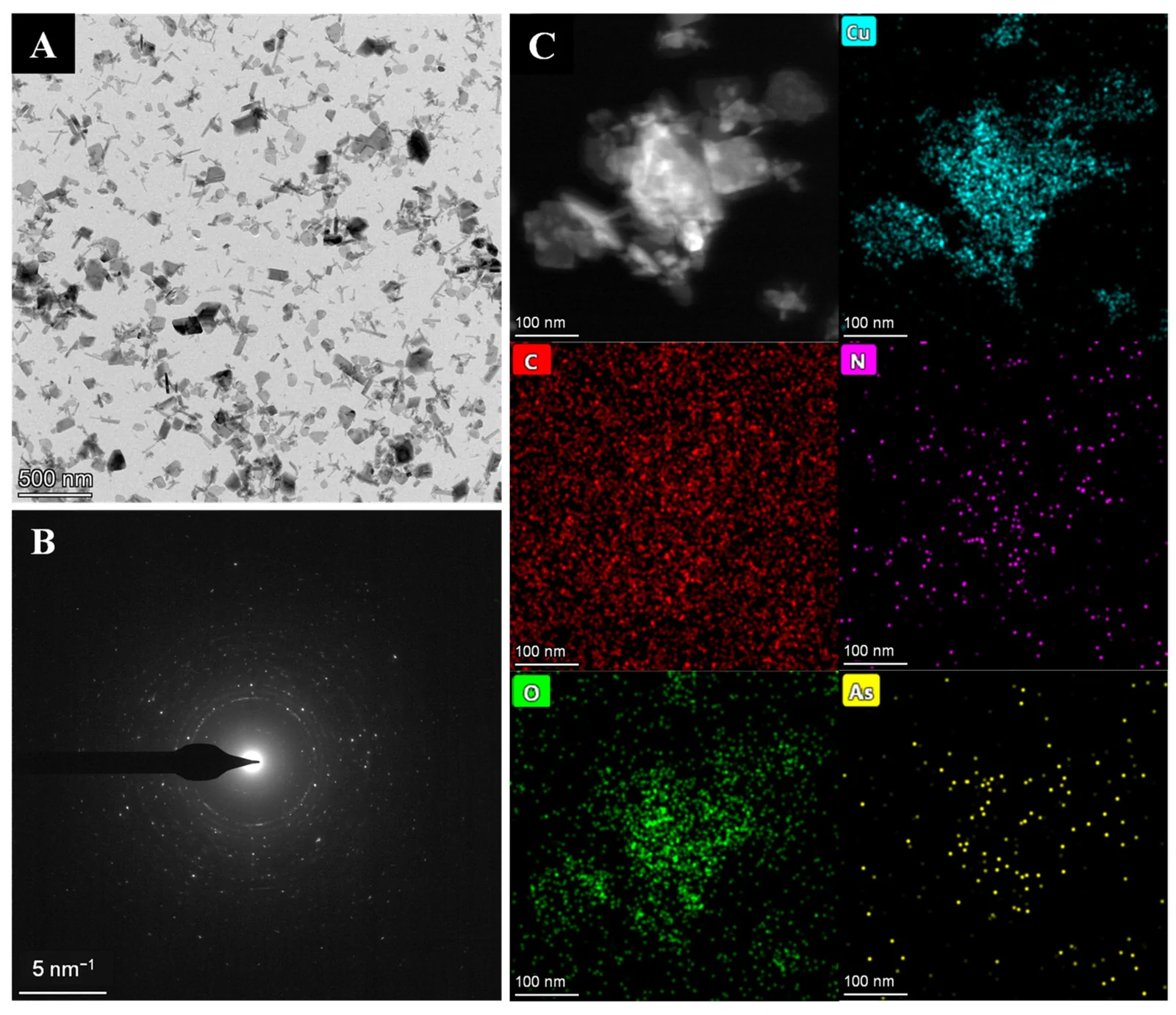
(**A**) TEM image, (**B**) SAED pattern, and (**C**) EDX mapping images of the NCuzyme after incubation with As(V).

To further investigate the possible enhancement mechanisms, the NCuzyme was characterized following the incubation with As(V). First, as shown in Figure 4A, there is no significant change in the morphology of the material after the As(V) addition. The SAED pattern in Figure 4B also shows no changes compared with that of NCuzyme (Figure 1C), indicating that the material’s original crystal structure was not influenced by As(V). The FT-IR spectra (Figure S3A) further demonstrate that incubation with As(V) did not disrupt the framework structure of NCuzyme. The EDX images (Figure 4C) display a small amount of As uniformly distributed across the surface of NCuzyme. Furthermore, as shown in Figure S3B, a signal peak corresponding to As appears in the full-scan spectrum of NCuzyme after incubation with As(V). And the high-resolution As 3d spectrum (Figure S3C) displays a single fitted peak (45.31 eV) attributed to the As(V), further confirming the presence of As(V) on the NCuzyme surface.

The results of the zeta potential measurements (Figure S4) show that after the addition of As(V), the zeta potential of NCuzyme decreased from −28.4 mV to −50.8 mV, indicating that the absorption of As(V) significantly increases the density of negative charges on the material’s surface. Upon adding the cationic substrate TMB, the zeta potentials in the system without As(V) and with As(V) are −22.7 mV and −31.1 mV, respectively. This change may be due to the positively charged TMB neutralizing the surface negative charge. However, the zeta potential of the system with As(V) remains significantly lower than that of the system without As(V). These results suggest that As(V) increases the negative charge on the material’s surface, thereby enhancing the electrostatic attraction to the positively charged TMB molecules, which helps to boost the local substrate concentration.

**Figure 5:**
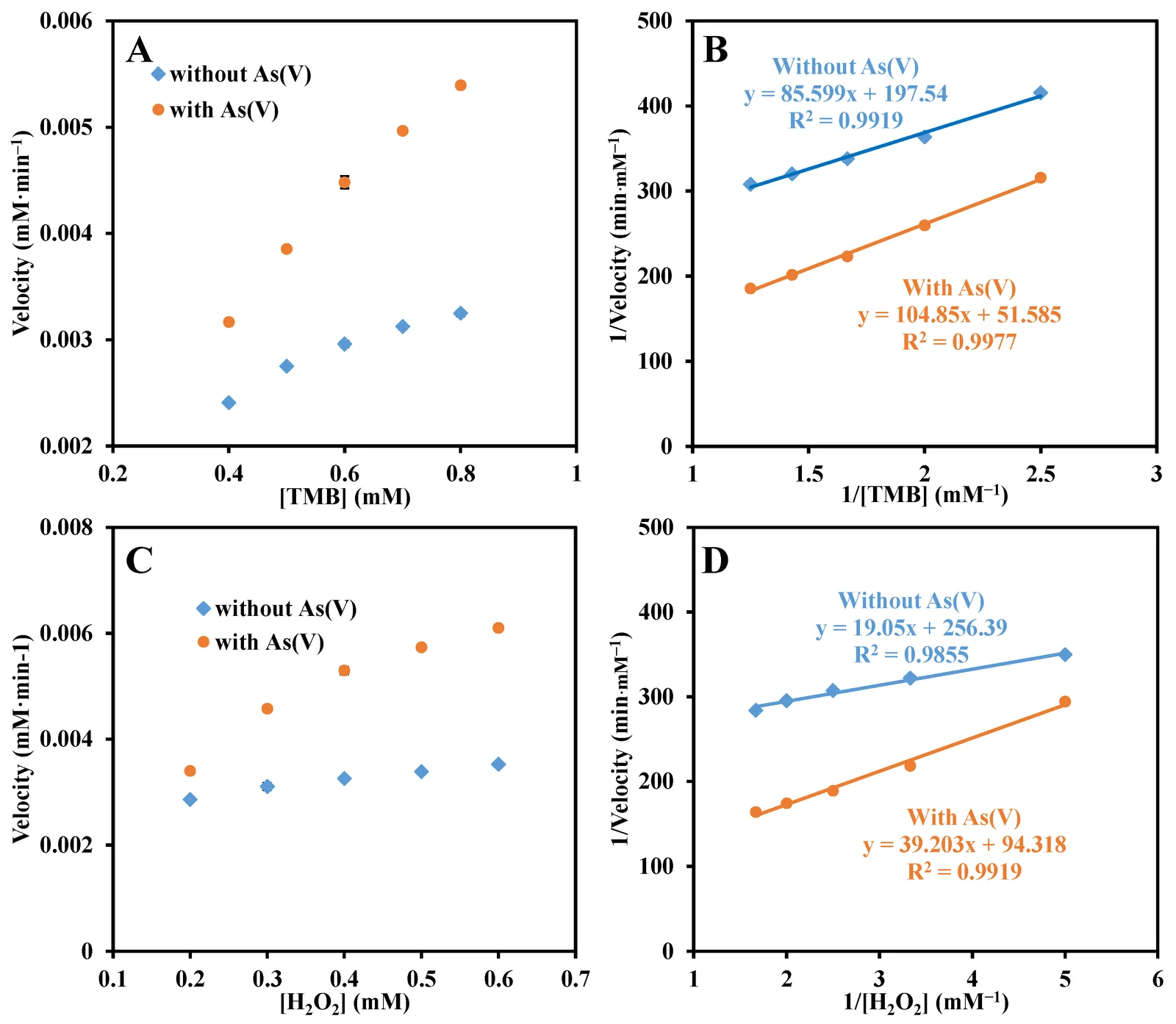
Reaction velocity of (**A**) TMB and (**C**) H_2_O_2_ with varied concentrations catalyzed by NCuzyme with and without incubation with As(V). (**B**,**D**) Corresponding double-reciprocal plots.

The enhancement mechanism was further investigated through steady-state kinetic experiments. Under optimized assay conditions, kinetic parameters were determined by fitting the Michaelis-Menten equation (Figure 5A,C) and the corresponding double-reciprocal curves (Figure 5B,D) after varying the concentrations of the substrates TMB and H_2_O_2_, respectively. As shown in Table 3, upon addition of As(V), the *V*_max_ values of NCuzyme for both TMB and H_2_O_2_ increased, indicating enhanced catalytic rates for these substrates. In addition, XPS results (Figure S3D) show that the proportion of Cu(I) increased after As(V) treatment, indicating that the valence state of Cu changed, which may affect the catalytic conversion process of the substrate. In summary, the possible mechanism by which As(V) enhances the POD-like activity of NCuzyme can be described as follows. First, the adsorption of As(V) increases the density of negative charges on the surface of NCuzyme, thereby enhancing its electrostatic attraction to the positively charged TMB and increasing the local substrate concentration; second, the presence of As(V) increases the proportion of Cu(I) and improves the catalytic conversion efficiency, accelerating the substrate’s catalytic transformation. The combined effect of these two factors ultimately results in a significant improvement of the catalytic activity of NCuzyme.

## 3. Materials and Methods

### 3.1. Chemicals

Please find the detail information of “Text S1 Chemicals” in Supplementary Material.

### 3.2. Apparatus

Please find the detail information of “Text S2 Apparatus” in Supplementary Material.

### 3.3. Preparation of NCuzyme

Compared with conventional hydrothermal methods [28], our synthesis avoids organic solvents and high-temperature/high-pressure conditions. Using a simple and green method, the NCuzyme was prepared. First, 0.126 g of MEL was heated and dissolved in ultrapure water (10 mL) to yield a colorless and transparent solution. This solution was then added to a beaker containing 10 mL of CuCl_2_·2H_2_O (0.341 g), and stirred at 500 rpm for 5 min at 45 °C on a multi-position magnetic stirrer. After centrifugation at 10,000 rpm (10,080 *g*) for 3 min, the blue precipitate was collected and washed with ultrapure water three times. Finally, the suspension of the collected material was prepared by dispersing it in 1 mL of water and stored at 4 °C before use.

### 3.4. Optimization of Experiment Conditions

Using the difference value of absorbance (∆A = A − A_0_, A and A_0_ are the absorbance of the system with and without arsenic, respectively) of the reaction system before and after the addition of As(V) as an indicator, the single-factor control variable method was employed to optimize the synthesis conditions of NCuzyme (molar ratio of Cu(II) and MEL, synthesis temperature and time) and detection conditions, including the dilution factor of NCuzyme, type of buffer and pH, concentrations of TMB and H_2_O_2_, reaction temperature, and reaction time. In brief, 150 μL of NCuzyme suspension (diluted 40 times), 150 μL of TMB (10 mM), 150 μL of H_2_O_2_ (10 mM), and 1050 μL of H_2_O were added to a 2 mL centrifuge tube sequentially and was reacted in an oven at 35 °C for 5 min. Using a UV-Vis spectrophotometer, the absorption spectra in the range of 500–800 nm were recorded, and the absorbance at 652 nm was measured. In addition, 150 μL of As(V) solution (1 μg/mL) was added to replace 150 μL of H_2_O in the above reaction system, and the absorbance of the system (652 nm) after the addition of As(V) was recorded.

### 3.5. Study on Steady-State Kinetics of NCuzyme

Under optimized conditions, the steady-state kinetics of NCuzyme POD-like activity were studied through changing the TMB and H_2_O_2_ concentrations in the reaction system and recording the absorbance of the solution (652 nm) after the reaction. The kinetic parameters (*K*_m_ and *V*_max_) were calculated according to the Lineweaver–Burk double reciprocal curve (1/*V* = *K*_m_/(*V*_max_ × [*S*]) + 1/*V*_max_). Among them, *K*_m_ represents the Michaelis constant, *V* represents the initial reaction rate, *V*_max_ represents the maximum reaction rate, and [*S*] represents the concentration of substrate (TMB or H_2_O_2_).

### 3.6. Determination of As(V)

Under the optimized detection conditions, the absorbance of the system at different As(V) concentrations was measured, and the calibration curve was constructed based on the relationship between concentration and absorbance. First, 150 μL of NCuzyme suspension (diluted 40 times), 150 μL of different concentrations of As(V) solutions, 150 μL of TMB (15 mM), 150 μL of H_2_O_2_ (10 mM), and 900 μL of H_2_O were added sequentially into centrifuge tubes (2 mL) and reacted in an oven at 35 °C for 6 min. Then, the UV-Vis absorption spectra in the range of 500–800 nm were measured, and the absorbance (652 nm) was recorded. The LOD value was calculated according to the formula LOD = 3*σ*/*k*, where *σ* is the standard deviation of (*n* = 11) blank solution (excluding the analyte), and k is the slope of the calibration curve.

### 3.7. Interference Resistance and Selectivity Study

To investigate the selectivity of the colorimetric method based on NCuzyme for As(V) detection, different metal ions like As(III), Mn(II), Fe(III), Co(II), Cu(II), Ni(II), Zn(II), Cd(II), Ag(I), Cr(VI), Pb(II), Na(I), Mg(II), Al(III), K(I), and Ca(II), were added to the detection system and analyzed. Furthermore, the above interfering ions and As(V) were added simultaneously to the reaction system with concentrations of 0.02 mM for As(V), Fe(III), and Pb(II), and 0.1 mM for the remaining interfering ions. The other detection procedures were followed the same as above.

### 3.8. Colorimetric Detection of As(V) in Real Sample

The dried rice sample (from Yonghui Supermarket (Chongqing, China)) was ground into powder. The sample powder was accurately weighed (15 mg), dissolved in water (15 mL), and ultrasonicated for 30 min. The obtained rice sample solution was filtered through a 0.22 μm membrane. For the above samples, As(V) at different concentrations (25, 50, and 75 μg/L) was spiked for recovery tests.

## 4. Conclusions

Based on the significant boosting effect on the POD-like activity of NCuzyme by As(V), a simple and sensitive colorimetric method for the detection of As(V) has been established. This method has excellent selectivity for As(V) with a LOD of 3.35 μg/L. Its application in the determination of As(V) in rice yielded good recovery rates (93.3–102.9%). Furthermore, mechanistic studies indicate that the enhancement of NCuzyme’s POD-like activity by As(V) may be primarily attributable to two factors: (1) the enhancement of electrostatic adsorption of TMB through an increase in surface negative charge, thereby raising the local substrate concentration; and (2) a significant improvement in catalytic conversion efficiency. The present work not only provides a facile and rapid analytical approach for As(V) detection, but also extends the application of Cu-based materials with enzyme-like activity in the field of heavy metal ion determination.

## Figures and Tables

**Table 1:** Comparisons on the colorimetric detection of As(V) with previously reported methods.

Material	Target	LOD (μg/L)	Linear Range (μg/L)	Ref.
CoOOH nanoflakes	As(V)	3.72	4–500	[29]
UMnzyme	As(V)	2.4	12.5–250	[22]
α/CyD-AgNPs	As(III)	12.5 μg/mL	20–500 μg/mL	[30]
AuNPs@MB	As(V)	–	1–100	[31]
Tar-AuNPs-Fe(III)	As(III)	21	0–1000	[18]
NCuzyme	As(V)	3.35	4–150	This work

**Table 2:** Detection of As(V) in real samples (*n* = 3).

Sample	Added (μg/L)	Found (μg/L)	Recovery Rate (%)	RSD (%)
Rice	0	0	–	–
	25	23.3	93.3	1.2
	50	50.1	100.1	0.8
	75	77.2	102.9	2.3

**Table 3:** Comparison of the kinetic constants of NCuzyme with and without incubation with As(V).

Catalysts	Substrate	*K*_m_ (mM)	*V*_max_ (10^−8^ M·s^−1^)
NCuzyme	TMB	0.43	8.44
NCuzyme + As(V)		2.00	32.30
NCuzyme	H_2_O_2_	0.074	6.50
NCuzyme + As(V)		0.42	17.70

## Data Availability

The original contributions presented in this study are included in the article. Further inquiries can be directed to the corresponding author.

## Supplementary Materials

The following supporting information can be downloaded at https://www.mdpi.com/article/10.3390/molecules31193494/s1. Text S1: Chemicals; Text S2: Apparatus; Figure S1: XPS spectra of NCuzyme: (A) C 1s, (B) N 1s, and (C) O 1s; Figure S2: (A) UV-Vis absorption spectra of different reaction solutions; Effects of (B) molar ratio of Cu(II) to MEL, (C) synthesis temperature, (D) synthesis time, (E) NCuzyme dilution ratio, (F) TMB concentration, (G) H_2_O_2_ concentration, (H) type of buffers, (I) pH of H_2_O, (J) reaction temperature, and (K) reaction time on the detection of As(V); Figure S3: (A) FT-IR spectra of NCuzyme before and after incubation with As(V); XPS spectra of NCuzyme after incubation with As(V): (B) survey spectrum, (C) As 3d and (D) Cu 2p; Figure S4: Zeta potential of (1) NCuzyme, (2) NCuzyme + As(V), (3) NCuzyme + TMB + H_2_O_2_, and (4) NCuzyme + TMB + H_2_O_2_ + As(V).
