# Supplementary material for: A Colorimetric Strategy for Arsenic(V) Determination in Rice via Boosting the Peroxidase-like Activity of NCuzyme
Source: Molecules. 2026 Oct 1;31(19):3494. doi: 10.3390/molecules31193494 (PMC13666798; doi:10.3390/molecules31193494)
Supplement: Supplementary file 1 [file molecules-31-03494-s001.zip › molecules-4554738-supplementary.pdf]

Supplementary material

A colorimetric strategy for arsenic(V) determination in rice via boosting the peroxidase-like activity of NCuzyme

Lei Xu, Mao-Ling Luo, Shan Qiu, Jing-Jing Dai, Huan Zhu, Feng-Qing Yang*

School of Chemistry and Chemical Engineering, Chongqing University, Chongqing 401331, China

Corresponding authors:

*Prof. Dr. Feng-Qing Yang, School of Chemistry and Chemical Engineering, Chongqing University, Chongqing 401331, China. E-mail: fengqingyang@cqu.edu.cn.

Table of contents

Supplementary Chemical and Apparatus

Text S1. Chemicals

Text S2. Apparatus

Supplementary Figures

Figure S1. XPS spectra of NCuzyme: (A) C 1s, (B) N 1s, and (C) O 1s.

Figure S2. (A) UV-Vis absorption spectra of different reaction solutions; Effects of (B) molar ratio of Cu(II) to MEL, (C) synthesis temperature, (D) synthesis time, (E) NCuzyme dilution ratio, (F) TMB concentration, (G) H_2O_2 concentration, (H) type of buffers, (I) pH of H_2O, (J) reaction temperature, and (K) reaction time on the detection of As(V).

Figure S3. (A) FT-IR spectra of NCuzyme before and after incubation with As(V); XPS spectra of NCuzyme after incubation with As(V): (B) survey spectrum, (C) As 3d and (D) Cu 2p.

Figure S4. Zeta potential of (1) NCuzyme, (2) NCuzyme + As(V), (3) NCuzyme + TMB + H_2O_2, and (4) NCuzyme + TMB + H_2O_2 + As(V).

Supplementary Chemical and Apparatus

Text S1. Chemicals

As(V) standard solution (As(V)，1001 μg/mL) and As(III) standard solution (As(III)，1001 μg/mL) were purchased from Beijing North Weiye Metrology Research Institute (Beijing, China). Cadmium chloride hemi(pentahydrate) ($CdCl_2·5/2H_2O$, 98%), cobalt chloride hexahydrate ($CoCl_2·6H_2O$, AR), and nickel(II) chloride hexahydrate ($NiCl_2·6H_2O$, 99%) were obtained from Macklin Co., Ltd. (Shanghai, China). Cu(II) chloride dihydrate ($CuCl_2·2H_2O$, AR) was purchased from Shanghai Aladdin Biochemical Technology Co., Ltd. (Shanghai, China). The 3,3′,5,5′-tetramethylbenzidine (TMB, >98.0%) was obtained from Adamas-beta (Shanghai, China). Sodium chloride (NaCl, ≥99.5%), potassium chloride (KCl, ≥99.5%), hydrochloric acid (HCl, AR), H_2O_2 (30% (w/w) in water), zinc sulfate heptahydrate ($ZnSO_4·7H_2O$, ≥99.5%), silver nitrate ($AgNO_3$, GR), aluminum nitrate nonahydrate ($Al(NO_3)_3·9H_2O$, ≥99%), manganese (II) sulfate monohydrate ($MnSO_4·H_2O$, ≥99%), magnesium sulfate heptahydrate ($MgSO_4·7H_2O$, 99%), lead nitrate ($Pb(NO_3)_2$, ≥99%), potassium dichromate ($K_2Cr_2O_7$, AR), and MEL (≥98%) were purchased from Chengdu Chron Chemicals Co., Ltd. (Chengdu, China). The 2-morpholinoethanesulfonic acid (MES, 98%) and N-(2-hydroxyethyl) piperazine-N′-2-ethanesulfonic acid (HEPES, 99.5%) were obtained from Meryer Biochemical Technology Co., Ltd. (Shanghai, China). Sodium hydroxide (NaOH) was purchased from Shanghai

Titan Scientific Co., Ltd. (Shanghai, China). Tris(hydroxymethyl)methyl aminomethane (Tris, 99.9%) was obtained from BBI Life Sciences Co., Ltd. (Shanghai, China). Sodium acetate anhydrous (NaAc, AR) was purchased from Chongqing Chuandong Chemical (Group) Co., Ltd. (Chongqing, China). Calcium chloride ($CaCl_2$, ≥96%) was obtained from Tianjin Damao Chemical Reagent Factory (Tianjin, China). Ferric chloride ($FeCl_3$, 99%) was obtained from Beijing MREDA Technology Co., Ltd. (Beijing, China). All experimental water was purified by a water purification system (ATSelem 1820A, Chongqing Antson Environmental Protection Equipment Co., Ltd.) (Chongqing, China).

Text S2. Apparatus

Transmission electron microscopy (TEM) images and element distribution analysis were recorded using a Talos F200S electron microscope (Thermo Fisher Scientific Inc. Brno, Czech Republic) working at 200 kV, which is equipped with an energy dispersive X-ray spectrometer (EDX). X-ray diffraction (XRD) patterns were obtained using an X'pert Powder diffractometer (Malvern Panalytical Ltd., Alemlo, Netherlands) with secondary beam graphite monochromated Cu Kα radiation. Fourier transform infrared spectroscopy (FT-IR) spectra were taken on a Nicolet iS10 spectrometer (Shimadzu, Kyoto, Japan). X-ray photoelectron spectrometry (XPS) was recorded on a PHI5000 Versaprobe system using monochromatic Al Ka radiation (1486.6 eV), and the obtained binding energies were referenced to the C 1s line set at 284.8 eV

(Thermo Fisher Scientific Ltd., Waltham, MA, USA). Zeta potential was determined using NanoBrook Omni (Brookhaven Instrument, Holtsville, NY, USA). Ultraviolet-visible (UV-Vis) absorption spectra were recorded on a UV-8000S spectrophotometer (Shanghai Metash Instruments Co., Ltd., Shanghai, China).

Supplementary Figures

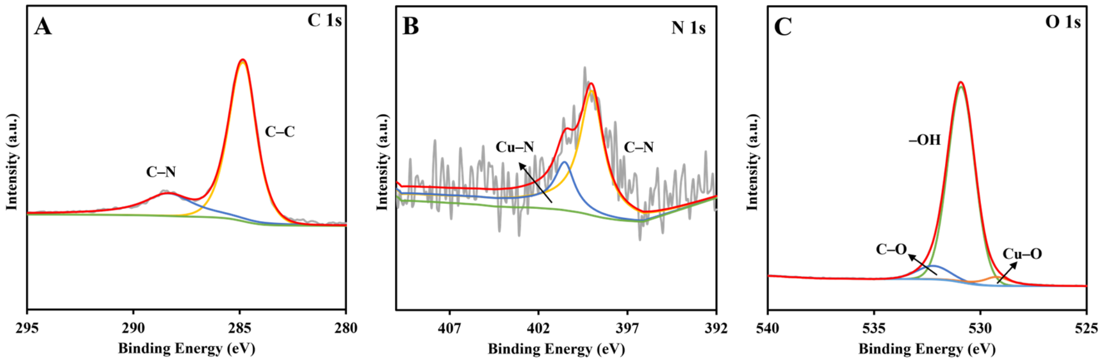

Figure S1. XPS spectra of NCuzyme: (A) C 1s, (B) N 1s, and (C) O 1s.

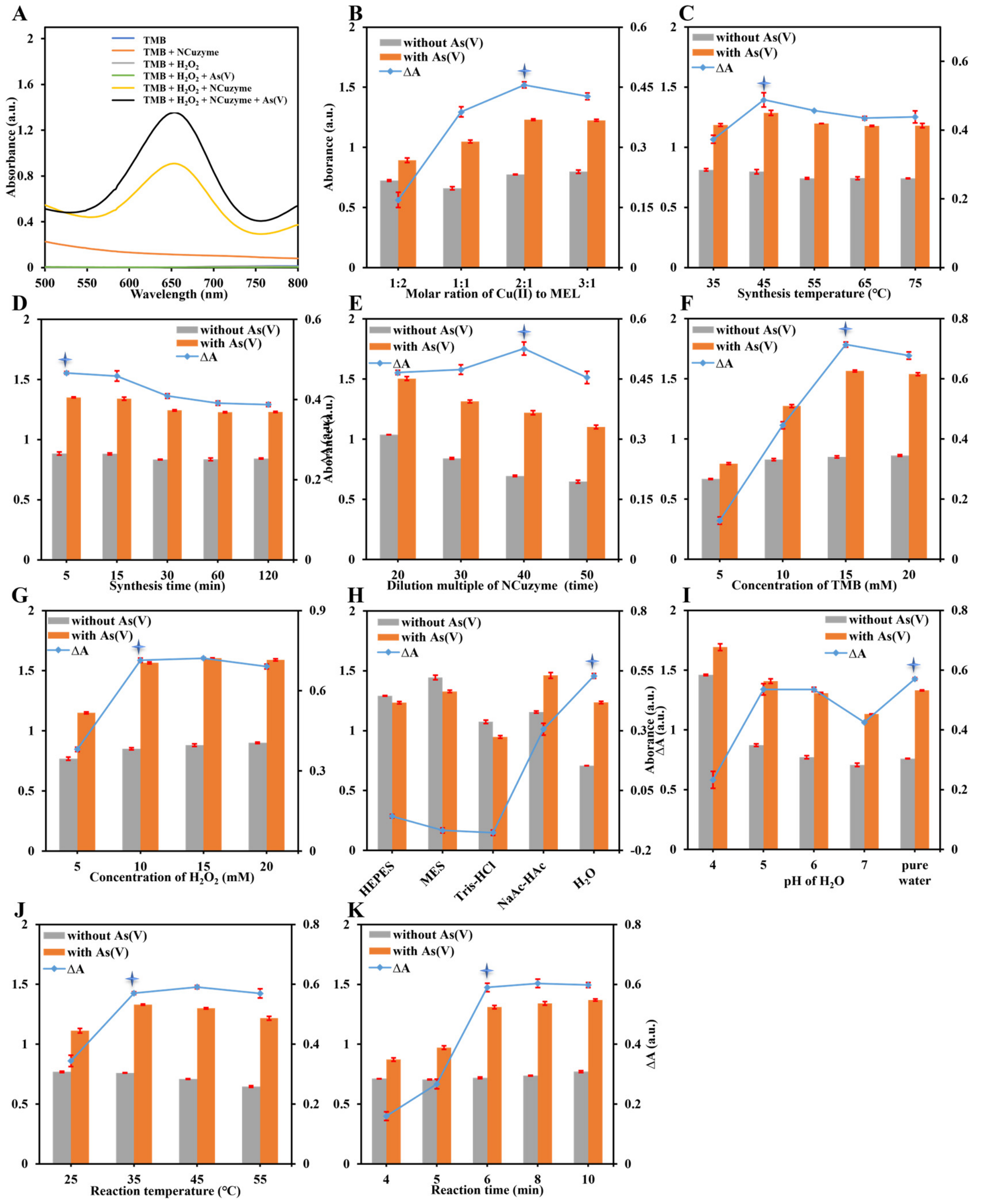

Figure S2. (A) UV-Vis absorption spectra of different reaction solutions; Effects of (B) molar ratio of Cu(II) to MEL, (C) synthesis temperature, (D) synthesis time, (E) NCuzyme dilution ratio, (F) TMB concentration, (G) H_2O_2 concentration, (H) type of buffers, (I) pH of H_2O, (J) reaction temperature, and (K) reaction time on the detection of As(V).

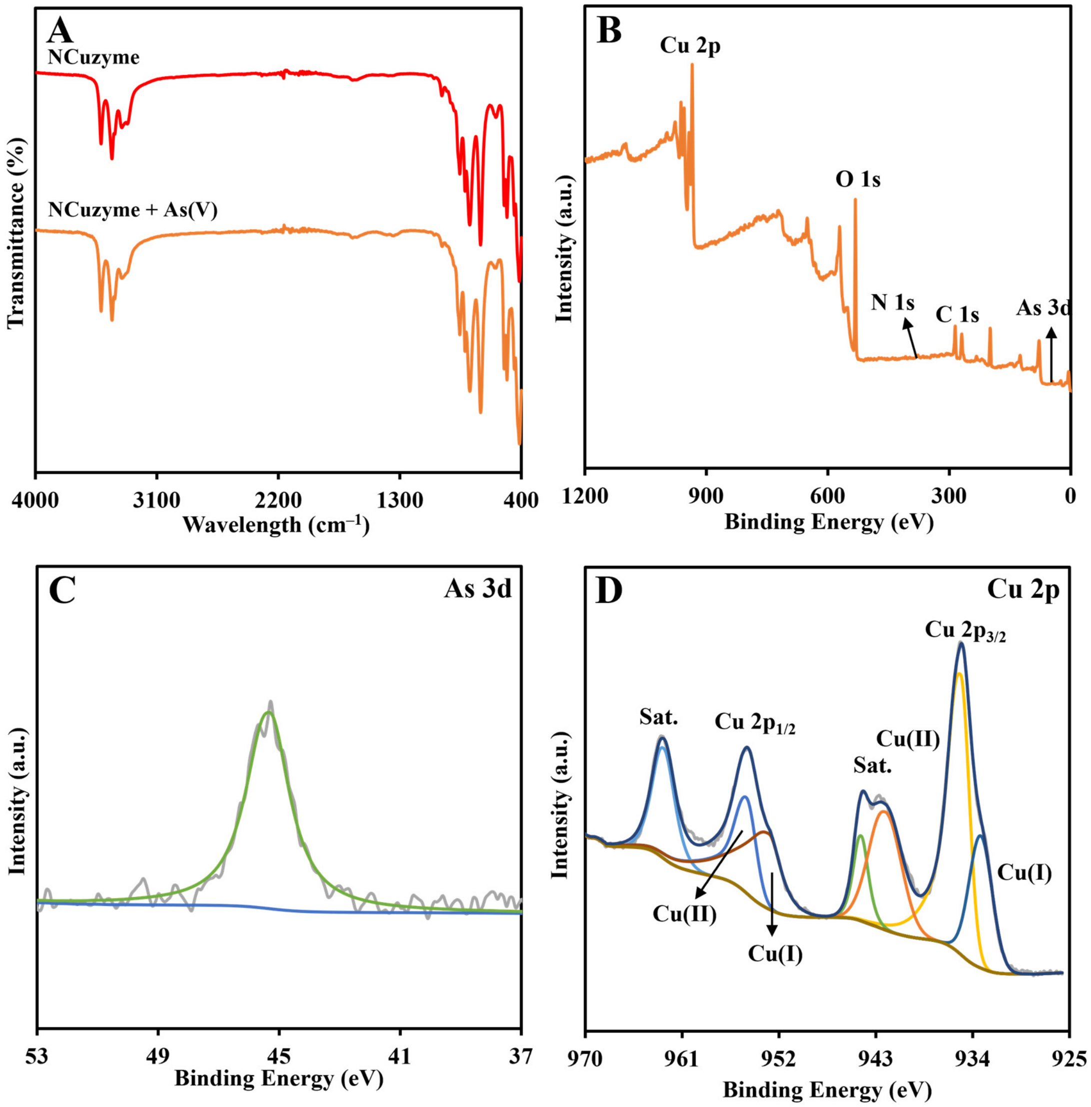

Figure S3. (A) FT-IR spectra of NCuzyme before and after incubation with As(V); XPS spectra of NCuzyme after incubation with As(V): (B) survey spectrum, (C) As 3d and (D) Cu 2p.

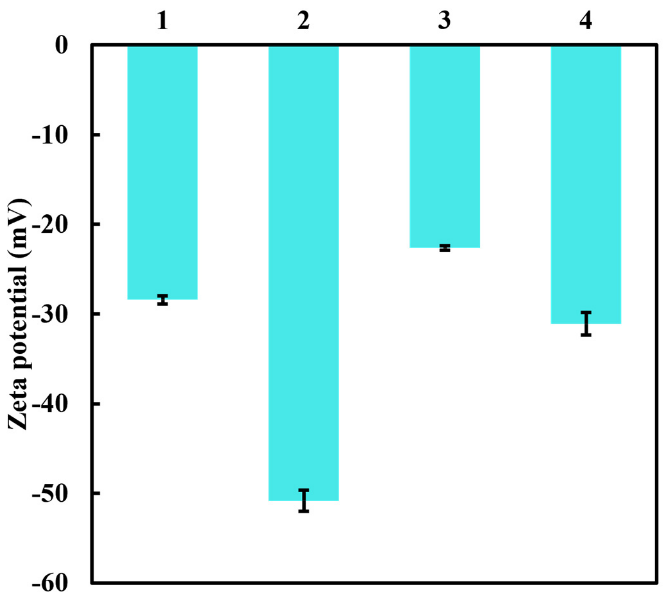

Figure S4. Zeta potential of (1) NCuzyme, (2) NCuzyme + As(V), (3) NCuzyme + TMB + H_2O_2, and (4) NCuzyme + TMB + H_2O_2 + As(V).
